# Compositional RL Agents That Follow Language Commands in Temporal Logic

**DOI:** 10.3389/frobt.2021.689550

**Published:** 2021-07-19

**Authors:** Yen-Ling Kuo, Boris Katz, Andrei Barbu

**Affiliations:** ^1^ CSAIL, MIT, Cambridge, MA, Unites States; ^2^ CBMM, MIT, Cambridge, MA, United States

**Keywords:** reinforcement learning, linear temporal logic, compositionality, zero-shot generalization, multi-task learning

## Abstract

We demonstrate how a reinforcement learning agent can use compositional recurrent neural networks to learn to carry out commands specified in linear temporal logic (LTL). Our approach takes as input an LTL formula, structures a deep network according to the parse of the formula, and determines satisfying actions. This compositional structure of the network enables zero-shot generalization to significantly more complex unseen formulas. We demonstrate this ability in multiple problem domains with both discrete and continuous state-action spaces. In a symbolic domain, the agent finds a sequence of letters that satisfy a specification. In a Minecraft-like environment, the agent finds a sequence of actions that conform to a formula. In the Fetch environment, the robot finds a sequence of arm configurations that move blocks on a table to fulfill the commands. While most prior work can learn to execute one formula reliably, we develop a novel form of multi-task learning for RL agents that allows them to learn from a diverse set of tasks and generalize to a new set of diverse tasks without any additional training. The compositional structures presented here are not specific to LTL, thus opening the path to RL agents that perform zero-shot generalization in other compositional domains.

## 1 Introduction

To reliably interact with humans in physical world, robots must learn to execute commands that are extended in time while being responsive to changes in their environments. This requires the robot to jointly represent the symbolic knowledge in language and the perceptual information from the environment as well as generalize to different commands and maps.

A popular representation to encode complex commands is linear temporal logic, LTL (Pnueli, 1977). Commands expressed in LTL encode temporal constraints that should be true while executing the command. Executing such commands is particularly difficult in robotics because integration is required between the complex symbolic reasoning that finds satisfying sequences of moves for an LTL command and data-driven perceptual capabilities required to sense the environment. While individual formulas can be learned by deep networks with extensive experience, we demonstrate how to compose together tasks and skills to learn a general principle of how to encode all LTL formulas and follow them without per-formula experience. We demonstrate how to integrate the learning abilities of neural networks with the symbolic structure of LTL commands to achieve a new capability: learning to perform end-to-end zero-shot execution of LTL commands.

**FIGURE 1:**
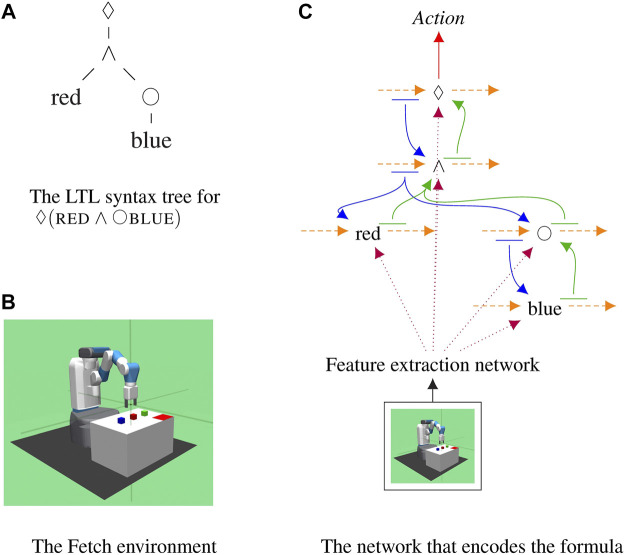
The proposed compositional recurrent network that encodes one formula, ⋄(red∧○blue). This formula corresponds to a command such as “Eventually move the red block into the tray and then move in the blue block.” **(A)** shows the syntax tree parsed from the formula. This tree provides the structure of the network. **(B)** is an example configuration of the Fetch domain, the Fetch robot is trained to move blocks of different colors in and out of the tray (i.e., the red rectangular area on the table). **(C)** shows the internal structure of the composed network.

Given a command represented as an LTL formula, our approach turns that formula into a specific recurrent deep network which encodes the meaning of that command; see Figure 1. The resulting network takes as input the current observations of the robot, processes it with a co-trained feature extraction network, and predicts which actions will satisfy the formula. This compositional approach ties together neural networks and symbolic reasoning allowing any LTL formula to be encoded and followed, even if it has never been seen before at training time.

**FIGURE 2:**
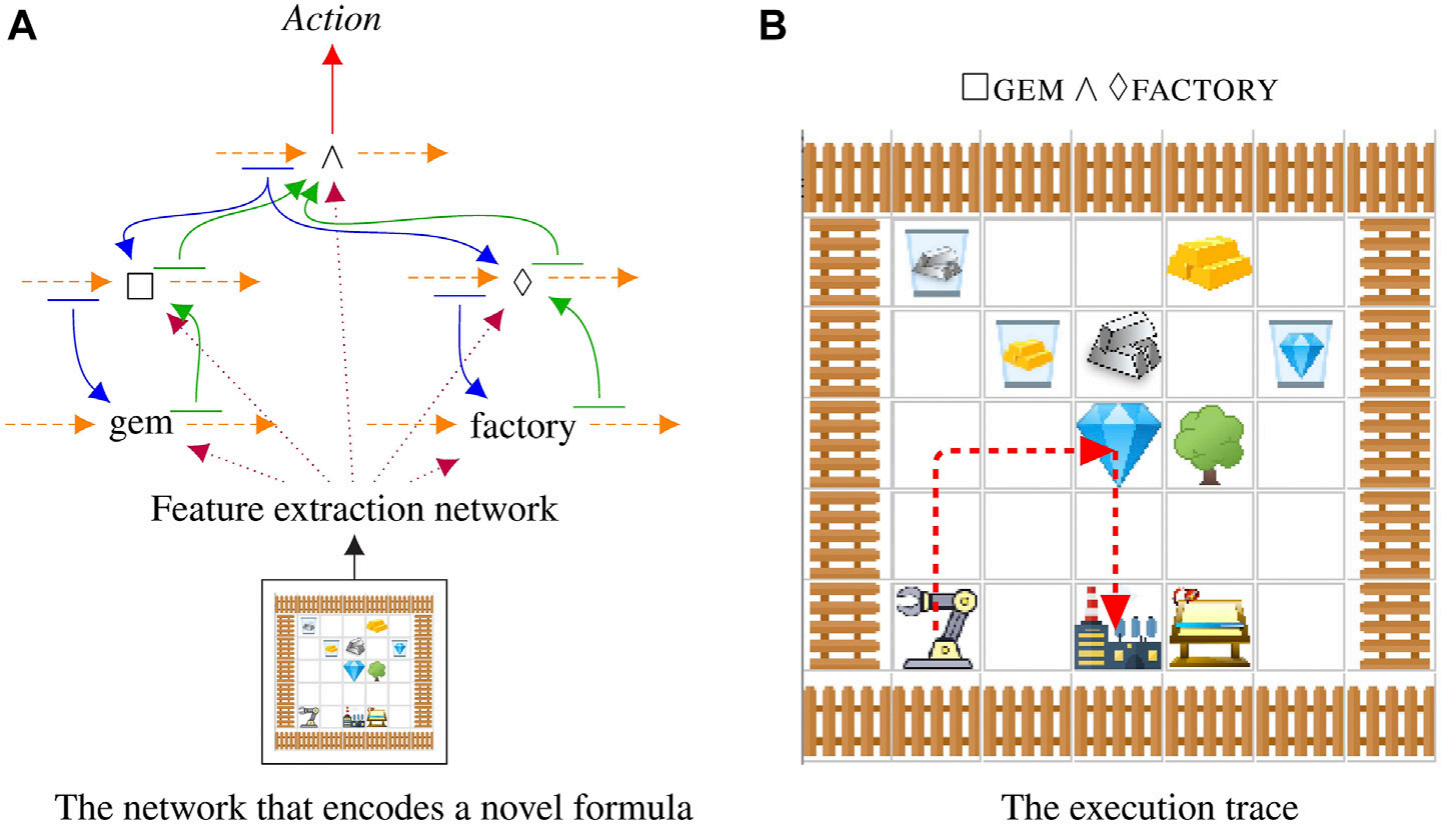
The model executing a novel command that was not shown at training time on a novel map. Maps and formulas are randomly generated. In this case the robot executes □gem∧⋄factory. **(A)** At testing time, the agent selects the trained sub-networks to compose a network to predict the action for each time step. **(B)** The robot successfully executes the command by going to the gem, picking it up, and, while holding on to the gem, going to the factory.

We show that the proposed compositional structure is compatible with many planning domains and algorithms, ranging from discrete state-action space with A2C agents to continuous state-action space with SAC agents. In three different domains, Symbol, Craft, Fetch, we demonstrate that the learned agent can execute never-before-seen formulas. The Symbol domain is more akin to Boolean satisfiability, where an accepting string must be generated for an LTL formula. The Craft domain is a simplified Minecraft introduced by Andreas et al. (2017) to test the integration with robotics; see Figure 2 for an example of the network in Figure 1 executing a command in the Craft domain. The Fetch domain is an environment adapted from OpenAI FetchPickAndPlace (Plappert et al., 2018) to test how the proposed approach can be extended to continuous state-action space. In all cases, we compare against baselines to demonstrate that each part of our model plays a key role in encoding temporal structures. All components of our networks are learned end-to-end from input observations to output actions, in a process that automatically disentangles the meaning of each sub-network allowing us to compose sub-networks together in novel ways.

In all of our experiments, we generate random LTL formulas and train an RL agent to follow those formulas. We find that as with SAT instances, random LTL formulas tend to be very easy and quite similar to one another. To that end, we develop a mechanism for generating hard and diverse LTL formulas. This is generally useful for other large-scale experiments on following commands that can be encoded as LTL formulas and for checking the accuracy and performance of LTL toolboxes.

This work makes the following contributions:

1. a trained deep network for following LTL commands in both discrete and continuous space,
2. end-to-end zero-shot execution of novel LTL commands,
3. co-training the feature extraction with the LTL controller, and
4. an investigation of the properties of random LTL formulas.

This work can be seen as a novel approach to 1) integrating perceptual information with symbolic knowledge and 2) composing the policies of multi-task reinforcement learning agents in a principled manner according to a particular logic. While we only discuss LTL here, this approach suggests how other logics might similarly be encoded to process the observed environment to create new models to zero-shot generalization in reinforcement learning. The best aspects of symbolic reasoning in robotics are compatible with deep networks when both are correctly formulated. We focus on command following, but in the future such methods could be used to monitor or describe behavior.

This manuscript starts by discussing the related research in learning and decomposing tasks expressed in LTL formulas. We then present the method to compose any given LTL formula as a policy network and explain how we can employ existing RL algorithms to train such policy networks. Next, we demonstrate the zero-shot generalization capability of the learned networks in both discrete and continuous domains. We conclude the manuscript with discussions about the pros and cons of the proposed approach as well as the future directions to extend this approach to make robots more reliable.

## 2 Related Work

The most related work to what we present here is in the area of multi-task learning and task composition. Previous work has learned finite state machines in conjunction with robot controllers (Araki et al., 2019). The focus in that prior work is on training extensively with one LTL formula and then following that formula; here we show how to zero-shot follow novel LTL formulas on new maps.

A related line of research shows how to accelerate learning of new LTL formulas by shaping rewards according to the structure of the formulas (Camacho et al., 2019). Here we provide no examples of novel formulas whereas this prior work requires tens of thousands to millions of training steps, in the Craft domain, for each new formula.

Compositions of LTL sub-formulas have been investigated before. Sahni et al. (2017) show how to compose together controllers for LTL formulas. The formulas considered are small: the largest is well below the mean size of our formulas. Only four formulas are tested thoroughly. Some zero-shot generalization is achieved to four formulas which have the same structure as those in the training set or which are carefully stitched together by hand given knowledge of their semantics. No general algorithm is given for how to encode an arbitrary LTL formula, nor is it possible to automatically train on a set of generated formulas.

LTL has been used to decompose tasks into sub-tasks, learn policies for each of the subtasks, and to improve the reliability and generalization capabilities of robots (Toro Icarte et al., 2018). Their approach must re-learn each subtask; in essence, the structure of the LTL formulas themselves plays no role. Here we show a more extreme approach where tasks encoded in LTL are composed together and directly followed without any task-specific training. Moreover, Toro Icarte et al. (2018) consider only 10 tasks which are paired with training data are considered, whereas here we execute thousands of new tasks. We adopt a variant of the Craft domain in Andreas et al. (2017). The Craft domain was originally developed for multi-task RL.

Recently, the structure of LTL formulas have been used to design the models (Araki et al., 2021; Vaezipoor et al., 2021). Similar to us, Vaezipoor et al. (2021) use the syntax tree to construct a graph neural network to interpret a formula. They assume an accurate event detector in both training and testing time to track the progress of the LTL formula. Here we only need an event detector at training time to provide reward signals and can predict actions at testing time without such information. Araki et al. (2021), on the other hand, decompose a formula into subgoals, safety propositions, and event propositions, and create a SMDP that learns options to achieve the subgoals as well as a meta-policy to compose the subgoals. Their approach requires retraining for a few iterations for any unseen formulas.

Safety-critical systems benefit from following constraints expressed as LTL formulas (Alshiekh et al., 2018). Shielded reinforcement learning prevents agents from entering states which violate constraints that can be expressed in LTL. This is critical for many real-world applications such as autonomous cars that can adapt to new environments. The approach presented here is complementary; it could provide an effective compositional shield that encodes new constraints. In the future, one might even be able to learn what constraints are required to shield a system. Several publications investigate combining Markov decision processes and Q-learning with constraints specified in LTL (Fu and Topcu, 2014; Sadigh et al., 2014; Wen et al., 2015).

Prior work has attempted to augment learning to satisfy constraints with policies derived from those constraints (Li et al., 2017; Li et al., 2018). This is a finer-grained analysis of the formulas than we perform here. While this speeds up learning, each formula requires significant training data.

## 3 Model

The model we introduce is presented in Figure 1. It is inherently compositional, i.e., the structure of the model reflects the parsing structure of the input LTL formula. In this section, we describe how the compositional network can be constructed. Then, we present the reward structure that encourages the agent to follow the LTL formulas. Finally, we discuss how to train this compositional network with two deep reinforcement learning algorithms: Advantage Actor-Critic and Soft Actor-Critic.

### 3.1 Composing LTL Formulas as Networks

Each LTL formula is parsed into a tree where the nodes are the operators or predicates; see Figure 1A. Operator and predicates are replaced by recurrent networks and are connected with one another according to the parse tree of the LTL formula. Every LTL formula generates a unique network that encodes the meaning of that specific formula.

The model is structured in such a way that knowledge flows between operators and predicates; see Figure 1C. Each operator and predicate in the formula is represented by a network, shown as a black node, selected from a trained collection of sub-networks. Sub-networks are RNNs which maintain hidden states over time, shown as orange arcs. Each sub-network takes as input features extracted from the observations of the robot using a co-trained network, shown in dotted purple lines. At every time step, the current state of all operators is decoded using a linear layer and passed to its children, shown in blue. This allows models to communicate information about the current sequence of steps to their children. The next state of each sub-network is decoded by a linear layer and passed to its parents, shown in green. This is the only form of communication between sub-networks. Parents let their children know about the current state sequence and children let the parent update their representations based on observations. Finally, the state of the root node is decoded by the actor and the critic into distribution over the value of the actions the robot can take at the current time step. Crucially, due to the compositional representation employed, novel formulas that were never seen at training time can be encoded and followed on novel configurations. This couples the power of deep networks to learn to extract features and engage in complex behaviors with knowledge about the structure of formulas, to perform zero-shot execution.

We use the intuition developed in Kuo et al. (2020a) that models which are composed of sub-networks can disentangle the meaning of words in a sentence without direct supervision. In other words, the agent is never told what ○ is supposed to mean as opposed to gem. Yet this can be done automatically, since from the definition of LTL formulas we understand that the computation required for every single operator or predicate should be the same. A learning method that attempts to find the most parsimonious explanation of the meaning of these shared sub-networks should then hone in on their meaning as components of LTL formulas by virtue of being forced to share computation when the definition of LTL formulas demands it. This intuition makes clear why the model presented here can recompose the sub-networks into new formulas: it has the capacity to execute recurrent computations, it shares computations in a way that the definition of LTL requires, and it is rewarded when it replicates the computations that are required to satisfy LTL formulas.

### 3.2 Reward Structure

At training time, we supervise the agent with random LTL formulas. Each formula is converted to a Buchi automaton using Spot (Duret-Lutz et al., 2016). Note that we only consider and evaluate LTL with finite traces (De Giacomo and Vardi, 2013) here, meaning that these automata are interpreted as having the semantics of finite automata. Each training episode proceeds with the agent taking a sequence of actions. The resulting world after taking each action is evaluated against the automaton. The predicates in the possible transitions of the automaton are evaluated. If the predicates hold, the agent is given a small, 0.1, reward. Staying in the same non-accepting state lowers the reward at a reward decay rate 0.8 for all three domains. If the predicates do not hold, the agent has violated the semantics of the LTL formula and it receives a large negative, −1, reward. If the predicates hold and the agent is in an accepting state, it receives a large positive, 1, reward. This reward structure encodes the notion that agents should follow an automaton which encodes a particular LTL formula, although agents do not have access to the automaton directly. We employ curriculum learning to sort generated formulas and provide shorter formulas first. Short formulas are more likely to accept more strings and their shallower corresponding models make error assignment more reliable.

### 3.3 Training RL Agents

In prior work, when learning two different policies, the networks for those two policies would be unrelated to one another. The compositional networks presented here can be seen as a principled way to share weights between these networks informed by the structure of LTL formulas. All operators and symbols share weights both within a formula and between updates, i.e., there is only a single model for ○ or gem.

We train the network using actor-critic methods. Actor-critic models learn a value function, the critic, that determines the score of a state, and a policy, the actor, which determines what to do at a given state. These could in principle be two separate networks. The critic network evaluates state, *s* at time *t*, $V_{v}\left(s_{t}\right)$ with learned parameters *v*. The actor network evaluates the effect of action, *a*, at time *t*, given a state $s_{t}$, $A_{\theta }\left(s_{t},a_{t}\right)$, with learned parameters *θ*.

#### 3.3.1 A2C-Based Agent for Discrete Space

In discrete state-action space, we employ an Advantage Actor-Critic (A2C) (Sutton et al., 2000; Mnih et al., 2016) agent. A2C models use the fact that the actor is computing an advantage, a change in value between two states, to estimate the actor using the value function of the critic. Practically, this means that a single network is required from which both the actor and the critic can be computed. The compositional model shown in Figure 1 is this shared network between the actor and the critic. The parameter updates of this network follow the standard methodology for training recurrent networks with A2C presented in Mnih et al. (2016).

#### 3.3.2 SAC-Based Agent for Continuous Space

In continuous state-action space, we employ a Soft Actor-Critic (SAC) (Haarnoja et al., 2018) agent. SAC is an off-policy deep RL algorithm in maximum entropy framework that improves exploration and robustness in continuous state-action space. The critic samples actions from a replay buffer to estimate the gradients. In the case of training compositional recurrent networks, instead of having one single replay buffer, we need to have separate replay buffers for different formulas as the structure of hidden states are different for different formulas. The parameter updates of this network follow the SAC algorithm presented in Haarnoja et al. (2018).

## 4 Experiments

We demonstrate the model in three domains. The first, Symbol, is designed to stress the symbolic reasoning abilities of RL agents. An LTL formula is provided to an agent which must immediately produce a satisfying assignment to that formula as a series of symbols. The second, Craft, is designed to stress the multi-task execution capabilities of RL agents in a robotic environment. An LTL formula and a map containing a robot are provided to an agent which must immediately find a sequence of moves that result in behavior of the robot that satisfies the LTL command. The third, Fetch, is designed to test the RL agents’ capabilities of executing sequences of actions in a continuous state-action space. An LTL formula and a 3D environment containing a robot are provided to the RL agent. The robot arms can move continuously in the 3D space where the agent needs to find a sequence of joint movements to manipulate the objects to satisfy the LTL command.

### 4.1 Training and Testing Setups

We generate various datasets to evaluate the proposed model and three ablations of the model.

For the two discrete domains, Symbol and Craft, we generated four datasets for each domain to include a diverse set of formulas in training and testing. A training dataset is first generated. Note that even in the case of the training set, our method has a significant advantage over prior work: our training set contains thousands to tens of thousands of formulas which are all learned, as opposed to learning one or a small handful of formulas. Then three test sets are generated of increasing difficulty. A test set which has roughly the same statistics as the training set in terms of formula length, i.e., 1–10 predicates with an average of 8, one which has 10–15 predicates, with an average of 13, and one which has 15–20 predicates with an average of 18. These test sets stress the generalization capabilities of the model and demonstrate that even when faced with formulas that are well beyond any that have been seen before in terms of complexity, the compositional nature of the model often leads to correct executions.

For the continuous Fetch domain, we similarly have shorter training formulas and longer testing formulas. Because there are only three objects in the Fetch domain, the difficulty of a formula is measured by the number of actions the robot needs to take and number of objects the robot must manipulate. The training set contains up to two events and the robot needs to manipulate up to two objects. The test sets contain the formulas the agent has not seen in the training set. The first test set has the same statistics as the training. The second test set is much harder. It requires the robot to carry out three or four events to move the objects.

### 4.2 Experiment Domains

#### 4.2.1 Symbol Domain

The Symbol domain is introduced here as a new challenge for multi-task LTL-capable agents. It removes the map and focuses on generating accepting strings for an LTL formula. The map can be a crutch for agents, e.g., crowded maps can have few paths making even random exploration efficient. The absence of particular resources on the map can also significantly simplify the problem, e.g., if there is no gem on the map, the agent can’t mistakenly pick up a gem. In the Symbol domain, given a fixed inventory of symbols, the agent predicts the assignment of symbols at each time step, a sequence of fixed length that will be accepted by the LTL formula.

In the experiments reported here, we use an environment that has five symbols and requires satisfying sequences of length 15. Changing the number of symbols does make the problem more difficult but not substantially so. This is because the approach presented here separately learns networks for each symbol making it robust to increasing the number of symbols; adding a symbol corresponds to adding a single sub-network.

Data generation is complicated by the fact that generating random LTL formulas produces uninteresting and easily satisfied instances. In a related domain, this is a well-known property of random instances of Boolean satisfiability, SAT, problems (Selman et al., 1996). We adapt solutions from this community to generating interesting collections of LTL formulas that are diverse and difficult to satisfy.

The generation process begins by sampling a formula from a uniform distribution over trees with one parameter: the prior distribution over the number of elements (predicates and symbols). This formula is converted to a Buchi automaton using Spot (Duret-Lutz et al., 2016). For a fixed *n*, the number of strings up to length *n* that the formula accepts is computed using a dynamic programming algorithm. This recursively computes the number of accepted strings for each time step, *N*. All formulas which have an acceptance ratio of more than 0.0001% are rejected as being too easy. Most randomly generated formulas tend to accept almost all strings. This provides hardness but does not guarantee variety.

In a second step, a second formula is generated as a function of the first formula. This uses a random process that picks a random subtree of the formula and replaces it with a new, also random, subtree. If the second formula is rejected, the generation process restarted based on the acceptance criteria above. If accepted, random accepted strings are sampled from the first formula and verified against the second. If more than 10% of strings that satisfy the first formula also satisfy the second, it is rejected as not diverse enough.

This may appear to be a rather laborious process, but without ensuring both hardness and diversity, we found that the generated formulas are uninteresting and fairly homogeneous in what strings or robot moves they accept. This is evidenced by the fact that without this process, there often existed a single string or sequence of robot moves which was accepted by the majority of generated formulas. After this more complex generation process, the resulting dataset is far more challenging, as is reflected in the experiments section by the low performance of baseline methods. The statistics of the generated datasets are shown in Table 1. The generation process is heavily biased against short formulas as they tend to be overly permissive and overlap with one another in meaning.

**TABLE 1:** Statistics of the generated formulas used in the Symbol domain and the Craft domain. Each test set is summarized using the mean and standard deviation of the number of symbols in the formula, total tree nodes in the parse of the formula, depth of the parse of the formula, and the states in the Buchi automata for those formulas.

Formula set	Symbols	Tree nodes	Tree depth	Automata states
Symbol (train)	3.28±0.49	9.14±1.16	4.49±0.96	4.21±0.52
Symbol (test, 1–10)	3.36±0.50	9.18±1.13	4.70±0.94	4.20±0.45
Symbol (test, 10–15)	4.64±0.77	13.05±1.49	6.04±1.14	5.32±1.75
Symbol (test, 15–20)	6.11±0.73	17.99±1.42	8.05±1.56	6.69±3.44
Craft (train)	2.76±0.63	7.94±1.49	4.35±1.04	3.40±0.77
Craft (test, 1–10)	2.78±0.64	7.83±1.46	4.22±0.98	3.37±0.74
Craft (test, 10–15)	4.33±0.82	12.94±1.54	6.62±1.05	3.73±0.96
Craft (test, 15–20)	6.06±0.75	18.14±1.31	7.94±1.37	4.44±1.96

#### 4.2.2 Craft Domain

**FIGURE 3:**
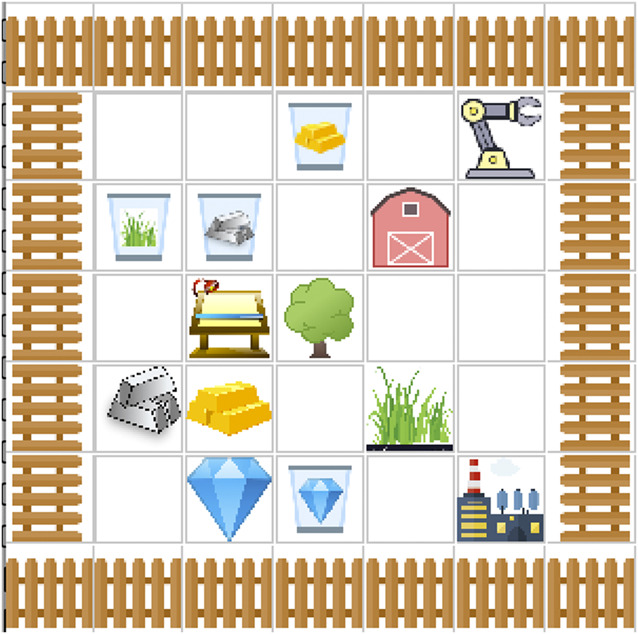
A 7×7 Craft domain example showing all the various elements available in the domain. The robot is in the top right corner and is controlled by the RL agent presented in this manuscript.

We use the Minecraft-like world already used by several prior multi-task learning reinforcement learning publications (Andreas et al., 2017; Toro Icarte et al., 2018). See Figure 3 for an example map that contains all of the elements of the Craft domain. It mixes together unmovable objects (trees, tool sheds, workbenches, and factories) with resources that can be manipulated (silver, grass, gold, and gems). We generate random Craft maps with random initial positions for the robot arm. The environment is 4-connected with an additional “use” action for the arm which picks up or drops the resource. The overall intent is that resources can be collected and manipulated through multiple processing stages resulting in a range of interesting tasks.

We extend this environment with a recycling bin so that agents can discard items. The four trash cans hold different resources: gold, grass, silver, and gems. This allows the agents to satisfy formulas that require owning an item for only part of the action.

Unlike in the Symbol domain, where most random formulas were trivially satisfied without our procedure to find hard and diverse LTL instances, in the Craft domain, most formulas are trivially unsatisfiable. Constraints on where the robot is at any one time and the fact that it must traverse the map step by step, make even basic formulas such as □GEM, the gem must always be held, unsatisfiable in all but the most fortunate situations. If the robot happens to be next to the gem, this formula can be satisfied by picking the gem up. If the robot is not next to the gem, no one action the robot can take will satisfy this formula.

We could employ rejection sampling and resample formulas until they can be satisfied. This would be wasteful and produce the same results as the following transformation. Since formulas are unsatisfiable in Craft largely because the robot does not have time to satisfy them, we introduce a transformation that gives the robot time. Whenever the robot needs to satisfy a predicate, we transform that predicate into “closer(*predicate*) ∪ *predicate,*” which states that the robot must get closer to its goal until it is reached. To make following this command feasible, the robot is given an additional feature which is the Manhattan distance to the predicate. This does not otherwise change the semantics of any of the formulas, nor does it make zero-shot generalization easier as the robot must still understand the command as a whole. The transformation also does not count toward the formula lengths we produce; it merely gives the robot some time. Indeed, this transformation actually makes learning harder because the agent does not receive any meaningful feedback if it runs out of time. It has no way of knowing if an episode failed because it could not reach a goal in time or because some constraint was violated. In the future, we intend to investigate how to make the causes of failure more transparent to the agents, thereby hopefully speeding up learning.

#### 4.2.3 Fetch Domain

We modify the OpenAI FetchPickAndPlace (Plappert et al., 2018), an environment which is used to evaluate several reinforcement learning algorithms in continuous state-action space, to make it compatible with multi-task learning. In our Fetch domain, there are three blocks of different colors, red, blue, and green, on the table the robot can interact with. We generate random initial positions of the blocks. A corner of the table is marked with the red rectangle to indicate the tray area. The goal of the robot is to move the blocks in and out of the tray given the instructions specified as LTL formulas. See Figure 1 for an example of table configuration and the robot.

We use three predicates in this environment. Each predicate represents one block. If a predicate is true, it indicates that the corresponding block should be in the tray, otherwise, outside of the tray. This can be viewed as multiple events to move the blocks. To specify the sequence of events, we use the SALT (Bauer et al., 2006) assertion language to synthesize the events and convert the assertions into LTL formulas. This allows us to specify the meaningful events and the number of events the robot should carry out easily. The sampling procedure works as follows. We first sample the number of events that should be in the assertion. Then, we sample the event from the three predicates in the sequence of events. If a predicate is included in an earlier event, the one sampled for the later event should be negated. This means the robot can only move a block out of the tray only if that block has already been moved in the tray in the earlier events. Similarly, we also randomly sample the “imply” operator to connect two sets of events where the left-hand-side of imply needs to be true in an earlier event. This sampling procedure generates assertions such as “assert/red;!red;green/” (move the red block in the tray, move it out, and then move the green block in). The reward is given by the automaton converted from the sampled SALT assertions.

This environment is more challenging than the regular Fetch pick-and-place, and it is also harder to succeed here than in the Craft domain, as the robot needs to produce the correct event sequence and manipulate objects at the same time. To give the robot more time to satisfy a predicate, similar to the Craft domain, we also apply the transformation “closer(*predicate*) ∪ *predicate*” to indicate that the robot needs to move its gripper closer to the block it should interact with before it can move the block.

### 4.3 Generated Formulas

Four sets of formulas are generated for the experiments in the Symbol and the Craft domains; see Table 1. The training set for each is generated with the same mechanism as (test, 1–10), i.e., containing formulas that have 1 and 10 symbols (i.e., operators and predicates). Training and test sets are randomly generated but verified to be disjoint. The test sets are used for testing zero-shot execution. To further demonstrate that this approach generalizes, we produce two additional test sets of even longer formulas than those seen in the training set. The 10–15 and 15–20 sets have longer formulas with, 10 to 15 symbols and 15 to 20 symbols, respectively.

For the Fetch domain, the number of events in an assertion corresponds to the size of the automaton. The training set contains eight formulas with up to two events; four require the robot to manipulate one object, another four require the robot to manipulate two objects. We have four test sets in the Fetch domain. The in-domain test set is the same as the training formulas. We have three out-of-domain test sets, two formulas in each set, that have no overlaps with training formulas. They have increasing difficulties as well. The first one has the same number of events as in training but refers to a different object and only one object needs to be moved. The second one is similar to the first but the robot has to move two objects. Finally, the test set with longer formulas which contains 3–4 events in which the robot needs to move one or multiple objects depending on the transitions to take in the automaton.

### 4.4 Hyper-Parameters

We consider only LTL formulas with finite traces (De Giacomo and Vardi, 2013) here, although the presented method is also compatible with executing LTL formulas in infinite time horizons. The interpretation of the results for LTL without a finite horizon is considerably more complex and requires new metrics because it must consider when a model fails, not just if it fails. In our experiments, if at the end of a rollout, the model is not in an accepting state, it is considered to have failed and given zero reward as if it had taken an action that violated the semantics of the LTL formula.

In all domains, we use a gated recurrent unit, GRU (Cho et al., 2014), for each sub-network that represents an operator or predicate. A single linear layer per sub-network is always used as the decoder that transmits information from children to parents and a single linear layer transmits information from parents to children in the model structure.

While training, the next move is an action from a distribution over possible actions. While testing, a deterministic policy chooses the optimal move. We observed a significant performance drop if the agents chose deterministically at training time or stochastically at test time. The rest of hyper-parameters are different for different domains depending on if it is discrete or continuous state-action space.

#### 4.4.1 Symbol and Craft Domains

We train the Advantage Actor-Critic, A2C, model with RMSprop (Tijmen Tieleman, 2012) optimizer for both domains. Agents must explore the space of symbols and moves thoroughly since our data generation process ensures that the LTL formulas are strict and few operations result in accepting states. To encourage this, we set a higher entropy weight of 0.1 and set the reward decay, *γ*, to 0.9 during training. The hidden sizes for GRUs are 64 for both domains. The Symbol domain has only one hidden layer and the Craft domain has two hidden layers since it is significantly more complex. We perform rolloutes for 15 time steps at every iteration.

#### 4.4.2 Fetch Domain

We train the Soft Actor-Critic, SAC, model with Adam (Kingma and Ba, 2015) optimizer at initial learning rate to be $5\times 10^{-3}$. When exploring the policy in continuous space, the SAC model is sensitive to the reward magnitudes. We run a grid at scale 1–15 and set a reward scale 5 to exploit the reward signal. The replay buffer size is $5\times 10^{4}$ shared across all formulas. When sampling from replay buffer to update gradients, we make sure the agent only samples the memory generated with the current training formula.

The hidden size in this domain is 64 with only one hidden layer because the dimensions of the input observations are lower than the Craft domain. In the Fetch domain, the robot takes the distance between the gripper and the blocks, the rotation of blocks, and the distance between the blocks and the tray as input. After every gradient step, we use soft target update to update the target Q-network with the target smoothing coefficient set to 0.005. We perform rollouts for 100 time steps at every iteration, and set the reward decay (*γ*) to a higher value, 0.99, as moving objects to a target location takes more time in continuous space.

### 4.5 Baselines

Most similar to our approach, the LTL2Actions framework (Vaezipoor et al., 2021) also parses an LTL formula into a syntax tree to construct a network for multi-task learning. However, they assume that an accurate event detector is available at training and testing time to progress the active sub-formulas. Our method only uses an event detector at training time to check satisfaction of a formula in order to provide rewards but the agent does not have access to the event detector during testing. Because of the assumption of event detectors, we cannot directly compare our model to LTL2Actions. Instead, we test three baselines in all domains to understand the importance of each component we add to our model. The “no structure, no language” baseline takes the current state of the world and attempts to predict what action the agent should take next. It is a standard RL agent with vanilla recurrent network and does not observe the LTL formula at all. This is the performance an off-the-shelf agent would have if it only knows the environment but doesn’t know about the language command. The “no structure” baseline takes as input the LTL formula, learns an embedding of that formula into a single 32-dimensional vector, and then attempts to follow it. To create the language embeddings, each operator and predicate in the formula is encoded as a one-hot vector. Sequences of these one-hot vectors are passed to an RNN which is co-trained to produce the embedding of the formula, much like the CNN that produces an embedding of the Craft environment. The formula is provided to every sub-network just like the observations of the environment. This is the performance an off-the-shelf agent would have if it does not understand compositional structure of the formulas. The “no time” baseline is an ablation of our model; it is structured but is missing any recurrent connections, i.e., all symbols are feed-forward networks instead of GRUs. This is the performance an off-the-shelf non-recurrent RL agent would have because it cannot keep track of its progress through the formula.

### 4.6 Results

The performance of the Symbol domain is reported on formula sets of 10,000 training formulas, while the Craft domain, which is considerably slower, is reported on 4,000 training formulas; see Table 2. Formulas which are perfectly executed are reported as successes, any errors are considered a failure.

**TABLE 2:** The performance of our model finding satisfying actions for LTL formulas. Each column summarizes the average success rate and standard deviation over three runs for each test set.

	In domain	Out of domain		
	Length 1–10	Length 1–10	Length 10–15	Length 15–20
Symbol domain	-	-	-	-
No structure, no language	0.14 ± 0.03	0.16 ± 0.01	0.13 ± 0.02	0.10 ± 0.02
No structure	0.82 ± 0.15	0.84 ± 0.08	0.48 ± 0.08	0.27 ± 0.04
No time	0.05 ± 0.04	0.03 ± 0.02	0.02 ± 0.01	0.03 ± 0.02
Ours	0.98 ± 0.01	0.90 ± 0.01	0.57 ± 0.03	0.42 ± 0.04
Craft domain	-	-	-	-
No structure, no language	0.47 ± 0.03	0.51 ± 0.02	0.48 ± 0.08	0.44 ± 0.12
No structure	0.61 ± 0.07	0.62 ± 0.04	0.47 ± 0.10	0.44 ± 0.13
No time	0.24 ± 0.16	0.23 ± 0.07	0.18 ± 0.01	0.18 ± 0.03
Ours	0.82 ± 0.09	0.82 ± 0.13	0.72 ± 0.19	0.64 ± 0.08

“In domain” refers to training and testing on the same formulas, it has the same size as the set of training formula set. Note that this task is already far more complex than what existing methods can do as the model must learn to execute thousands of LTL formulas given the formula as input. “In domain” set has 98% accuracy for Symbol and 82% accuracy for Craft. Ablations of our method show that without the particular compositional structure imposed the performance is two-third for “no structure” and even lower for the other ablations. Every part of our model is critical to performance as ablating any part away hurts performance tremendously. No previous method can learn to execute such formulas.

We then test out-of-domain test sets, each contains 100 formulas, to demonstrate that our model can zero-shot execute new formulas. The performance on formulas that have similar statistics to the ones in the training degrades only slightly when testing on new formulas, and is shown in the second column of Table 2, “Out of domain (1–10).” This shows zero-shot generalization and indicates that in all cases our method generalizes well. Finally, two increasingly complex out of domain scenarios are tested. One in which has formulas that are 1.5 times as long, and one with formulas that are twice as long. Note the poor performance of the baseline methods and ablations; our method performs better.

**FIGURE 4:**
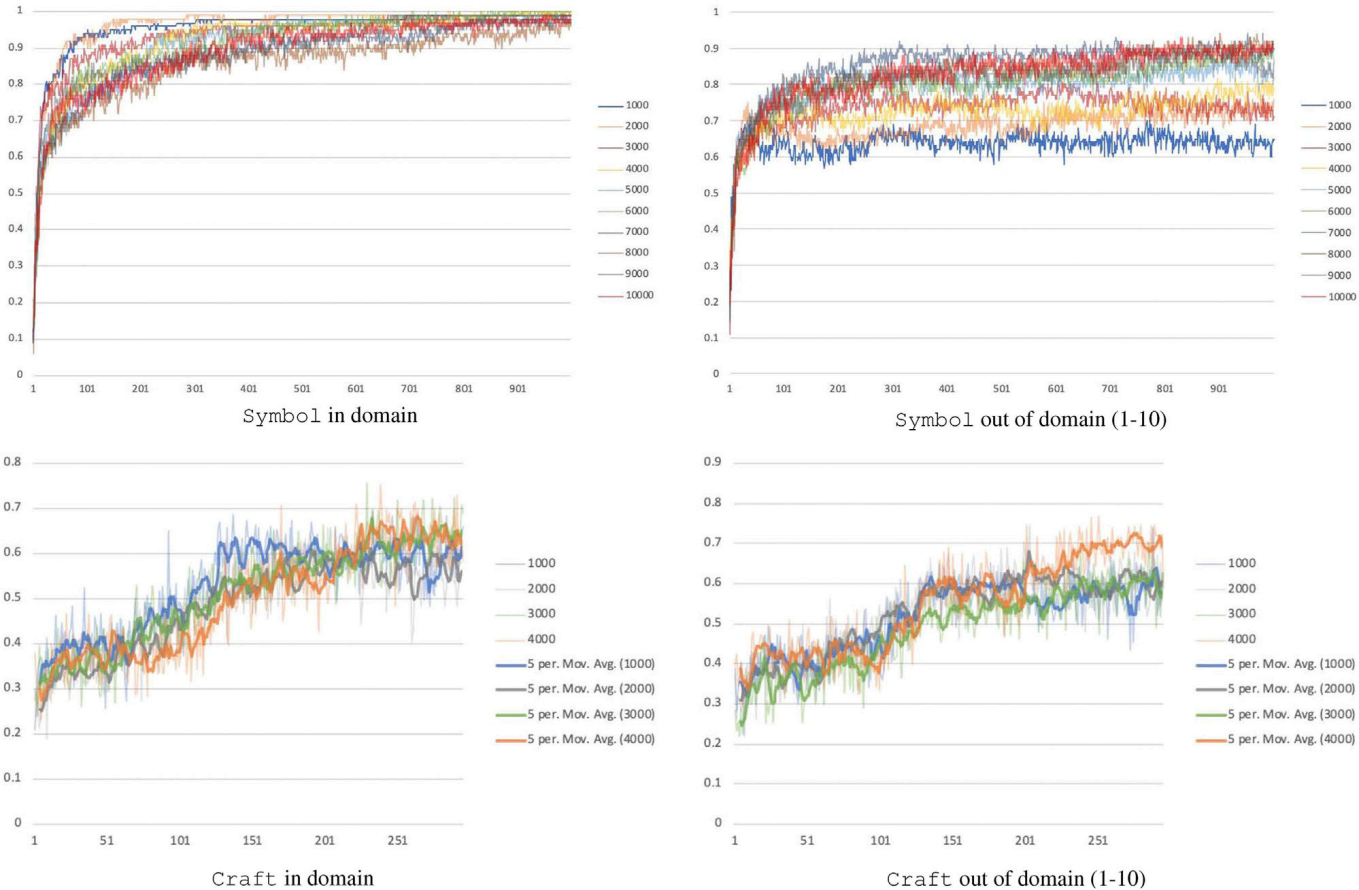
Performance of the model as the number of training formulas increases. The *x*-axis is the number of model updates performed, each unit is 200 updates for Craft and 500 updates for Symbol. Note that any minor error made while executing the formulas was considered a failure.

Absolute performance is a function of how many formulas our model is trained with. Several thousand formulas must be seen before generalization to new formulas and longer formulas is reliable, see Figure 4. The Symbol domain on 1,000 and 10,000 training formulas can reach similar in-domain performance but a 25% performance difference for out of domain (65 vs. 90% accuracy). As the number of training formulas increases, generalization improves. To extrapolate how many formulas would be needed for a given level of performance, we used a least squares fit to a logarithmic function, which had $R^{2}$ of 0.99. This predicts that performance would approach 100% at around 24,000 formulas. In other words, seeing 24,000 formulas that contain up to 10 predicates and operators is likely to be enough to perfectly generalize to all formulas of that length. To put this into context, with five symbols in the Symbol domain presented here, there are approximately $10^{10}$ formulas of length 10. This means that our network sees only one-millionth of all possible formulas before generalizing, not including its ability to generalize to longer formulas. Similarly in the Craft domain, 1,000 training formulas result in 59% accuracy out of domain, while 4,000 training formulas result in 70% accuracy out of domain. Together, these results show that our method learns to generalize formulas and to execute them zero-shot.

The performance of the Fetch domain is reported based on the number of events and blocks necessary to satisfy the command; see Table 3 for the success rate for 50 runs per formula. Our method can succeed 36% of time when a command can be satisfied by moving one block and 12% of time when it is required to move two blocks. Out of domain, when formula statistics are similar to the training set, our agent can generalize well for the commands involving one block but much worse for commands involving two blocks. This is because when the robot interacts with one object, it may move other objects to inconvenient locations, which makes it harder for the following events to be satisfied. For example, if the block to be picked up in the next event is accidentally pushed off the table, the robot has no way to recover and carry out the next event in sequence. In the future, we intend to investigate how to avoid these failure modes by providing feedback to the agent during command execution. Ablations of our method show that learning how to carry out commands in sequence in continuous domains is challenging, none of the existing methods can reliably execute commands and generalize to unseen formulas.

**TABLE 3:** The success rate of our model finding satisfying actions for LTL formulas in Fetch domain. The formulas for in-domain and out-of-domains are grouped by number of event and number of blocks the robot should interact with during the command execution.

	In domain		Out of domain		
	1–2 events	2 events	1–2 events	2 events	3–4 events
	1 blocks	2 blocks	1 block	2 blocks	1–2 blocks
Fetch domain	-	-	-	-	-
No structure, no language	0.05	0.00	0.04	0.00	0.00
No structure	0.05	0.01	0.05	0.00	0.00
No time	0.02	0.00	0.00	0.00	0.00
Ours	0.36	0.12	0.41	0.01	0.08

## 5 Conclusion

We developed a principled approach to encoding the temporal relationships required to follow LTL specifications in a compositional recurrent network. The pros and cons of the proposed approach are summarized as follows.

**Pros**: The compositional networks are cotrained with the features extraction network. This allows robots to learn the features that are useful for planning tasks. The compositional structure enables zero-shot generalization to unseen LTL formulas. Our experiments show that perfect generalization on both Symbol and Craft appears to be within reach.

**Cons**: Similar to other RL algorithms, training remains computationally expensive. Depending on the experiment and number of formulas, training the compositional networks took between 13 h and 3 days for Symbol and Craft and 6 days for Fetch on an Nvidia Titan X. Note that while relatively computationally intensive, such networks need only be trained once for any domain; due to their ability to zero-shot generalize to new formulas.

The proposed RL agents represent a new and powerful type of multi-task learning, where learning occurs on one set of tasks and generalizes to all others. Coupled with a semantic parser, this model could execute linguistic commands that refer to temporal relations; Wang et al. (2020) use the compositional model presented here to provide supervision to learn a LTL semantic parser. While we believe that such network architectures are useful for other logics, certainly for first-order logic as it is a subset of LTL, how to extend these ideas to other logics such as CTL^*^, modal logics, or second-order logics is unclear. This has a critical impact on language-driven robotics. Even if a model does not explicitly parse sentences into one of these formalisms, it must still internally possess the mechanisms that allow generalization in these domains. Theoretically characterizing the structures and conditions that enable neural networks to generalize to new problems that bear some compositional relationship to previously-seen problems is a new and exciting area of research.

Adapting to a 3D domain proved difficult, likely because exploration is significantly harder as the dimensionality of the planning problem increases. Adopting a hierarchical approach could ameliorate this problem. In the future, we also intend to investigate what additional priors, curricula, or training algorithms can speed up learning so we can extend to 3D domains in a more effective time frame.

As it stands, the reward function designed here to supervise the RL agent is powerful but lacks some critical feedback. For example, the agents do not know what went wrong; this is the failure mode we identified when interacting with multiple objects in the Fetch domain. Some feedback about the constraint that was violated could localize errors within particular sub-networks or feature extraction networks. This would be akin to telling someone that they should keep the blue block on the table because they need to pick it up next–clearly very useful information. Turning this intuition into a practical learning mechanism remains an open problem that would make the robots much more reliable.

## Data Availability

The original contributions presented in the study are included in the article/supplementary material, further inquiries can be directed to the corresponding author.

## References

[B1] Alshiekh M. Bloem R. Ehlers R. Könighofer B. Niekum S. Topcu U. (2018). “Safe Reinforcement Learning via Shielding,” in Thirty-Second AAAI Conference on Artificial Intelligence, New Orleans, Louisiana, USA (AAAI Press).

[B2] Andreas J. Klein D. Levine S. (2017). Modular Multitask Reinforcement Learning with Policy Sketches. In Proceedings of the 34th International Conference on Machine LearningVolume 70 (JMLR. org), 166–175.

[B3] Araki B. Li X. Vodrahalli K. DeCastro J. Fry M. J. Rus D. (2021). The Logical Options Framework, in Thirty-eighth International Conference on Machine Learning, Virtual.

[B4] Araki B. Vodrahalli K. Leech T. Vasile C.-I. Donahue M. D. Rus D. L. (2019). “Learning to Plan with Logical Automata,” in Proceedings of Robotics: Science and Systems, Freiburg, Germany (Robotics: Science and Systems Foundation).

[B5] Bauer A. Leucker M. Streit J. (2006). “SALT-structured Assertion Language for Temporal Logic,” in International Conference on Formal Engineering Methods (Springer), 757–775. 10.1007/11901433_41

[B6] Camacho A. Icarte R. T. Klassen T. Q. Valenzano R. McIlraith S. A. (2019). “LTL and beyond: Formal Languages for Reward Function Specification in Reinforcement Learning,” in Proceedings of the 28th International Joint Conference on Artificial Intelligence, 6065–6073.

[B7] Cho K. Van Merriënboer B. Bahdanau D. Bengio Y. (2014). On the Properties of Neural Machine Translation: Encoder-Decoder Approaches. in Eighth Workshop on Syntax, Semantics and Structure in Statistical Translation, Doha.

[B8] De Giacomo G. Vardi M. Y. (2013). “Linear Temporal Logic and Linear Dynamic Logic on Finite Traces,” in Twenty-Third International Joint Conference on Artificial Intelligence (Association for Computing Machinery), 854–860.

[B9] Duret-Lutz A. Lewkowicz A. Fauchille A. Michaud T. Renault E. Xu L. (2016). “Spot 2.0 — a Framework for LTL and *ω*-automata Manipulation,” in Proceedings of the 14th International Symposium on Automated Technology for Verification and Analysis (ATVA’16), Chiba, Japan, October 17-20, 2016.

[B10] Fu J. Topcu U. (2014). “Probably Approximately Correct MDP Learning and Control with Temporal Logic Constraints,” in Proceedings of Robotics: Science and Systems.

[B11] Haarnoja T. Zhou A. Abbeel P. Levine S. (2018). “Soft Actor-Critic: Off-Policy Maximum Entropy Deep Reinforcement Learning with a Stochastic Actor,” in International Conference on Machine Learning (PMLR), 1861–1870.

[B12] Kingma D. P. Ba J. (2015). “Adam: A Method for Stochastic Optimization,” in International Conference for Learning Presentations (ICLR), Ithaca, NY (arXiv.org).

[B13] Kuo Y.-L. Katz B. Barbu A. (2020a). “Deep Compositional Robotic Planners that Follow Natural Language Commands,” in International Conference on Robotics and Automation, Paris, France, 31 May-31 Aug. 2020 (IEEE). 10.1109/ICRA40945.2020.9197464

[B14] Kuo Y. L. Katz B. Barbu A. (2020b). “Encoding formulas as deep networks: Reinforcement learning for zero-shot execution of LTL formulas,” in International Conference on Intelligent Robots and Systems (IROS), Las Vegas, NV, October 24, 2020–January 24, 2021, 5604–5610. (IEEE). 10.1109/IROS45743.2020.9341325

[B15] Li X. Ma Y. Belta C. (2018). “A Policy Search Method for Temporal Logic Specified Reinforcement Learning Tasks,” in 2018 Annual American Control Conference (ACC), Milwaukee, WI, USA, 27-29 June 2018 (IEEE), 240–245. 10.23919/ACC.2018.8431181

[B16] Li X. Vasile C.-I. Belta C. (2017). “Reinforcement Learning with Temporal Logic Rewards,” in 2017 IEEE/RSJ International Conference on Intelligent Robots and Systems (IROS), Vancouver, BC, Canada (IEEE), 3834–3839. 10.1109/IROS.2017.8206234

[B17] Mnih V. Badia A. P. Mirza M. Graves A. Lillicrap T. Harley T. (2016). “Asynchronous Methods for Deep Reinforcement Learning,” in International conference on machine learning, 1928–1937.

[B18] Plappert M. Andrychowicz M. Ray A. McGrew B. Baker B. Powell G. (2018). Multi-goal Reinforcement Learning: Challenging Robotics Environments and Request for Research. arXiv preprint arXiv:1802.09464.

[B19] Pnueli A. (1977). “The Temporal Logic of Programs,” in 18th Annual Symposium on Foundations of Computer Science (IEEE), 46–57. 10.1109/SFCS.1977.32

[B20] Sadigh D. Kim E. S. Coogan S. Sastry S. S. Seshia S. A. (2014). “A Learning Based Approach to Control Synthesis of Markov Decision Processes for Linear Temporal Logic Specifications,” in 53rd IEEE Conference on Decision and Control (IEEE), 1091–1096. 10.1109/CDC.2014.7039527

[B21] Sahni H. Kumar S. Tejani F. Isbell C. (2017). “Learning to Compose Skills,” in Proceedings of NIPS 2017 Deep RL Symposium.

[B22] Selman B. Mitchell D. G. Levesque H. J. (1996). Generating Hard Satisfiability Problems. Artif. intelligence 81, 17–29. 10.1016/0004-3702(95)00045-3

[B23] Sutton R. S. McAllester D. A. Singh S. P. Mansour Y. (2000). “Policy Gradient Methods for Reinforcement Learning with Function Approximation,” in Advances in neural information processing systems, 1057–1063.

[B24] Tijmen Tieleman G. H. (2012). Lecture 6.5-RMSprop, Coursera: Neural Networks for Machine learningTech. Rep. University of Toronto.

[B25] Toro Icarte R. Klassen T. Q. Valenzano R. McIlraith S. A. (2018). “Teaching Multiple Tasks to an RL Agent Using LTL,” in Proceedings of the 17th International Conference on Autonomous Agents and Multiagent Systems), 452–461.

[B26] Vaezipoor P. Li A. Icarte R. T. McIlraith S. (2021). Ltl2action: Generalizing LTL Instructions for Multi-Task Rl. Thirty-eighth International Conference on Machine Learning, Virtual.

[B27] Wang C. Ross C. Kuo Y.-L. Katz B. Barbu A. (2020). “Learning a Natural-Language to LTL Executable Semantic Parser for Grounded Robotics,” in Conference on Robot Learning (CoRL) (arXiv:2008.03277).

[B28] Wen M. Ehlers R. Topcu U. (2015). “Correct-by-synthesis Reinforcement Learning with Temporal Logic Constraints,” in 2015 IEEE/RSJ International Conference on Intelligent Robots and Systems (IROS), Hamburg, Germany (IEEE), 4983–4990. 10.1109/IROS.2015.7354078

